# Non-pharmacological sleep interventions for pediatric cancer patients and survivors: a systematic review protocol

**DOI:** 10.1186/s13643-021-01724-3

**Published:** 2021-06-04

**Authors:** Peter L. Stavinoha, Ineke M. Olsthoorn, Maria C. Swartz, Sara Nowakowski, Stephanie J. Wells, Rachel S. Hicklen, Irtiza Sheikh, Hannah J. Jang

**Affiliations:** 1 Division of Pediatrics, University of Texas MD Anderson Cancer Center, 1515 Holcombe Blvd, Houston, TX 77030 USA; 2 School of Psychology, Fielding Graduate University, 2020 De La Vina St, Santa Barbara, CA 93105 USA; 3 Department of Medicine, Baylor College of Medicine & VA HSR&D Houston Center of Innovation, Michael E. DeBakey VA Medical Center, TMC - McGovern Campus, 2450 Holcombe Blvd, Houston, TX 77021 USA; 4 Research Medical Library, University of Texas MD Anderson Cancer Center, 1515 Holcombe Blvd, Houston, TX 77030 USA; 5 University of California, San Francisco Medical Center, Institute for Nursing Excellence, 2233 Post Street, Ste. 201, San Francisco, CA 94115 USA

**Keywords:** Non-pharmacological interventions, Pediatric cancer, Quality of life, Sleep

## Abstract

**Background:**

Sleep disturbances constitute a common complication in pediatric cancer patients and survivors and are frequently severe enough to warrant treatment. Suboptimal sleep has been associated with decreased emotional well-being and cognitive functioning and increased behavioral problems. Standardized guidelines for non-pharmacological sleep interventions for adults with cancer exist, but no standard of care intervention or standard guidelines are available to guide such intervention in pediatric cancer patients and survivors. Therefore, effective behavioral interventions for improving sleep quality need to be identified. The objective of the review is to evaluate the effect of non-pharmacological sleep interventions on sleep quality in pediatric cancer patients and survivors.

**Methods:**

The review will consider studies that include children and adolescents between 0 and 18 years diagnosed with cancer or who have a history of cancer who have non-respiratory sleep disturbance. We will include experimental and quasi-experimental studies evaluating non-pharmacological interventions such as psychological interventions, technical/device interventions, interventions targeting physical activity, and complementary and alternative medicine interventions (e.g., yoga, massage, music). Interventions involving medications, ingestible supplements, products purported to work through absorption, and medical devices will be excluded. Primary outcome will be sleep quality as measured by methods including retrospective ratings, daily sleep diary, and validated questionnaires. Secondary outcomes will include total sleep time, sleep onset latency, wake after sleep onset, daytime sleepiness, and daytime sleep duration (naps) as measured by retrospective ratings, daily sleep diary, validated questionnaires, and/or actigraphy. Databases will include MEDLINE (Ovid), EMBASE (Ovid), Cochrane Library, CINAHL (Ebsco), and PsycINFO (Ovid) and will be queried from database inception to present. Two reviewers will independently screen all citations, full-text articles, and extract data. The study methodological quality will be assessed using Joanna Briggs Institute (JBI) critical appraisal tools. Data will be extracted and findings pooled and synthesized using a meta-aggregation approach via the JBI System for the Unified Management, Assessment, and Review of Information (SUMARI). If feasible, we will conduct random effects meta-analysis. Additional analyses will be conducted to explore the potential sources of heterogeneity (e.g., methodological quality, study design, outcome measures).

**Discussion:**

This systematic review will synthesize and consolidate evidence on existing non-pharmacological interventions to improve sleep in pediatric cancer patients and survivors. Findings may help inform practitioners working with pediatric cancer patients and survivors experiencing sleep disturbances and is intended to identify gaps and opportunities to improve methodical quality of further non-pharmacological sleep intervention research in this population toward developing an eventual standard of care.

**Systematic review registration:**

PROSPERO CRD42020200397.

**Supplementary Information:**

The online version contains supplementary material available at 10.1186/s13643-021-01724-3.

## Background

Improved treatment methods for pediatric cancer have resulted in five-year survival rates of 80% and higher across childhood cancer types [1], and as a result, the importance of maximizing quality of life in this population has been increasingly recognized [2]. Specifically, quality of life can be negatively affected by a range of late effects of cancer, which may be physical, cognitive, emotional, or behavioral in nature [2]. In both pediatric cancer patients and survivors, sleep disturbances are a common complication [3], affecting quality of life [4], and are frequently severe enough to warrant treatment [5]. Sleep disturbances have been considered to negatively impact resilience in children [6] and can complicate coping with the various late effects of cancer in children and adolescents. As such, identifying and addressing sleep problems through sleep interventions may have an important role in maximizing quality of life in this population [7].

Sleep problems meeting criteria for sleep disorders occur at a significant rate in the pediatric cancer population. Specifically, sleep disordered breathing, also known as sleep apnea, occurs in a significant number of pediatric brain tumor survivors [8], while in children diagnosed with leukemia, insomnia has been frequently reported [9].

Sleep apnea, a respiratory sleep disorder, is not the focus of this review. Instead, the focus is on non-respiratory sleep problems manageable without medical device or medication and within the purview of allied health providers (e.g., nursing, psychology). Indeed, beyond diagnosable sleep disorders, a range of sleep disturbances have been observed in the pediatric cancer population, which frequently start after the initiation of treatment [9], and are more pronounced than in healthy children [10]. Sleep disruptions have been observed in hospitalized pediatric cancer patients [11], as well as in children and adolescent cancer patients living at home [12]. Furthermore, Russo and colleagues [3] found that in pediatric cancer survivors more than 5 years post-diagnosis, 44.6% of parents indicated their child to have a sleep disturbance. In line with this finding, other studies suggest sleep disturbances can become chronic in pediatric cancer, persisting years after the diagnosis into adulthood [5]. Excessive daytime sleepiness has been suggested to be the most commonly reported consequence of sleep disturbance in pediatric cancer [7, 9]. Excessive daytime sleepiness may result from insufficient sleep, fragmented sleep, or an enhanced sleep drive [13]. Reduced sleep times, increased wakefulness during the night, and lowered sleep efficiency have indeed been reported across pediatric cancer types [10].

Several models have been proposed to explain the onset and persistence of sleep problems in pediatric cancer patients and survivors, including the Sleep Disturbances in Pediatric Cancer Model (SDPCM) [14] and a behavioral model explaining sleep disruptions in pediatric cancer survivors [15]. The SDPCM proposes that cancer-related factors including the diagnosis and the treatment (e.g., medication, radiation, chemotherapy, or surgery) impact psychosocial, environmental, and biological factors, which, in turn, disrupt sleep [14]. Psychosocial consequences may involve family stress, reduced activity, parent limit setting, depression, and anxiety, while environmental factors may include hospitalization, noise, an irregular day schedule, and nighttime caretaking. Biological consequences contributing to sleep disruption may include factors such as pain, fatigue, nausea, increased urinary frequency, or endocrinological alterations [14]. Once sleep disturbances emerge, the disturbed patterns are proposed to persist due to perpetuating factors, including physiological changes due to cancer or the continuation of altered sleep habits (e.g., napping, increased time in bed, irregular sleep schedules). Furthermore, sleep problems may be further exacerbated by hyperarousal or increased stress or frustration around sleep [15].

According to the American Academy of Sleep Medicine, healthy sleep patterns have a vital role in maximizing health in the pediatric population and involve appropriate duration, timing, quality, and regularity of sleep [16]. For instance, sleep has been associated with several health-related outcomes, including general, cardiovascular, metabolic, mental, and developmental health. Additionally, sleep has been related to immunologic function and several performance measures [16]. In the general pediatric population, suboptimal sleep has been associated with decreased emotional well-being, including increased levels of distress [17], anxiety, depression, irritability, moodiness, and emotional lability. Behavioral problems have been reported as well, including overactivity, noncompliance, oppositional behavior, reduced impulse control, and risk-taking behavior [7]. Finally, cognitive functioning can be impacted by sleep disturbances as well, including executive functions, school performance [18], attention [7, 19], and processing speed [20].

The common occurrence and persistence of sleep problems in the pediatric oncology population and their potential impact on health and quality of life highlight the relevance of identifying behavioral sleep interventions aimed at ameliorating sleep disturbances. For instance, introducing families to behavioral strategies prior to starting with sleep medication may enhance long-term outcomes by stimulating healthy sleep habits in an earlier stage [14]. Additionally, research suggests that parents are more receptive to behavioral interventions compared to pharmacological treatment [14], and pharmacotherapy may not be the treatment of choice given the long trajectory of cancer treatment and the possible development of tolerance to the medications [21].

In the adult cancer population, cognitive behavioral interventions [22], exercise [23], and complementary and alternative methods [24] have been shown useful for improving sleep. Indeed, a practice guideline was published to address sleep disturbances in adults diagnosed with cancer in Canada [25], yet no comparable guideline or standard of care intervention is available for pediatric oncology [7]. Similarly, while psychological interventions [26], exercise [27], and complementary and alternative paradigms [28] have all undergone systematic review in pediatric oncology, sleep has not been a target of these interventions or reviews of their efficacy and, to the best of our knowledge, no current or underway systematic reviews exist on this topic.

The objective of the review will be to evaluate the effect of nonpharmacological sleep interventions on sleep quality in pediatric cancer patients and survivors.

## Methods

The proposed systematic review will be conducted in accordance with the Joanna Briggs Institute methodology for systematic reviews of effectiveness evidence studies [29] and the present review protocol adheres to the Preferred Reporting Items for Systematic Review and Meta-Analysis Protocols (PRISMA-P) statement [30] (see checklist in Additional file 1). The protocol is registered with PROSPERO, the international prospective register for systematic reviews, ID CRD42020200397. Any amendments to this protocol will be documented and published alongside the results of the systematic review.

### Eligibility criteria

Studies will be selected according to the following criteria: participants, interventions, comparators, outcomes, and types of studies.

### Participants

The review will consider studies that include children and adolescents diagnosed with cancer or who have a history of cancer. Children and adolescents will be between birth and 18 years at the time of sleep intervention. Children and adolescents may be at any stage of treatment or survivorship, and intervention may be offered in any setting (e.g., home, hospital, rehabilitation center, clinic, academic medical center). The target of intervention will be improving sleep in children and adolescents with cancer or a history of cancer, and targets for intervention may include patients, caregivers, family, and healthcare providers as long as the focus of intervention and outcomes include improving sleep in the pediatric cancer patients and survivors.

### Interventions

We are defining non-pharmacological interventions as methods including technical procedures, devices, rehabilitation paradigms, psychological and behavioral interventions, and complementary and alternative medicine interventions. Details will be gathered including mode of delivery, agent of delivery, timing, and frequency/duration of delivery. Interventions of interest include, but are not limited to, those summarized in Table 1.

**Table 1:** Examples of nonpharmacological interventions for sleep

Technical/non-invasive devices	Physical	Psychological	Complementary and alternative medicine
Activity/sleep trackers	Structured exercise programs	Cognitive therapy	Aromatherapy
Computer programs/phone apps	Physical rehabilitation	Psychotherapy	Music
Light manipulation	Lifestyle physical activity	Behavioral interventions	Yoga
Noise machines		Psychoeducational interventions	Massage
			Hypnosis

Pharmacological interventions targeting sleep that involve medications, ingestible supplements, products purported to work through absorption, or invasive medical devices (e.g., CPAP) will be excluded from this review.

### Comparators

This review will consider studies that compare the intervention to no intervention (usual care), sham or placebo intervention, and/or alternative intervention. The review will consider studies that include a comparison/control group, passive control group receiving no intervention, and within-group comparison to baseline with no control or comparator group.

### Outcomes

This review will consider studies that include the primary outcome of self-reported and/or caregiver/observer (proxy) reported outcomes of sleep quality. Primary outcome will be measured by methods including retrospective ratings, daily sleep diary, and validated questionnaires (e.g., Sleep Disturbance Scale for Children [31], Patient-Reported Outcomes Measurement Information System (PROMIS) Sleep questionnaires [32, 33], and Insomnia Severity Index [34]. Secondary outcomes will include total sleep time, sleep onset latency, wake after sleep onset, daytime sleepiness, and daytime sleep duration (naps) as measured by retrospective ratings, daily sleep diary, validated questionnaires (e.g., Epworth Sleepiness Scale) [35], and/or actigraphy or polysomnography.

### Types of studies

This review will consider both experimental and quasi-experimental study designs including randomized controlled trials, non-randomized controlled trials, before and after studies, and interrupted time-series studies. In addition, this review will also consider descriptive observational study designs including case series and individual case reports for inclusion. Only studies published in English will be included. No limitations will be imposed on publication date.

### Information sources and search strategy

The primary source of literature will be a structured search of five electronic databases (from inception onwards): MEDLINE (Ovid), EMBASE (Ovid), PsycINFO (Ovid), CINAHL (Ebsco), and Cochrane Library. Content experts and authors who are prolific in the field may be contacted. The literature searches will be designed and conducted by a qualified medical librarian (RSH). The search will include a broad range of terms and keywords related to sleep, sleep disturbance, pediatric population, cancer, and non-pharmacological interventions. Results will be limited to full-text articles published in the English language. Additionally, we will consider the reference lists of all included studies. A draft search strategy for MEDLINE (Ovid) is provided in Additional file 2.

### Study selection

Following the search, all identified citations will be collated and uploaded into EndNote X9 (Clarivate Analytics, PA, USA) and duplicates removed. Titles and abstracts will then be screened by two independent reviewers (IMO, IS) for assessment against the inclusion criteria for the review. For this step, the Rayyan [36] platform will be used to facilitate reviewer independence. Potentially relevant studies will be retrieved in full and their citation details imported into the Joanna Briggs Institute System for the Unified Management, Assessment and Review of Information (JBI SUMARI) (Joanna Briggs Institute, Adelaide, Australia) [37]. The full text of selected citations will be assessed in detail against the inclusion criteria by two independent reviewers (PLS, HJJ) using JBI critical appraisal tools. Reasons for exclusion of full-text studies that do not meet the inclusion criteria will be recorded and reported in the systematic review. Any disagreements that arise between the reviewers at each stage of the study selection process will be resolved through discussion, or with a third reviewer. A PRISMA flow diagram showing details of studies included and excluded at each stage of the study selection process will be provided [38].

### Assessment of methodological quality

Eligible studies will be critically appraised by two independent reviewers for methodological quality using standardized critical appraisal instruments from the Joanna Briggs Institute for experimental and quasi-experimental studies [29]. Authors of papers will be contacted to request missing or additional data for clarification, where required. Any disagreements that arise will be resolved through discussion, or with a third reviewer. The results of the critical appraisal will be reported in narrative form and in a table.

All studies, regardless of the results of their methodological quality, will undergo data extraction and synthesis (where possible).

### Data extraction

Data will be extracted from studies included in the review by two independent reviewers using the JBI SUMARI standardized data extraction tool. The data extracted will include specific details about the populations, study methods, interventions, and outcomes of significance to the review objective of assessing the impact of non-pharmacological interventions for sleep in pediatric cancer patients and survivors. Any disagreements that arise between the reviewers will be resolved through discussion, or with a third reviewer. Authors of papers will be contacted to request missing or additional data, where required.

### Data synthesis

Given the anticipated heterogeneity of studies, intervention methods, methodological design, and outcome measures likely to be encountered in this review, meta-analysis may not be feasible. In this case, the review will include a narrative synthesis of the interventional studies targeting improvement in sleep in pediatric cancer patients and survivors, incorporating structured guidance for narrative synthesis [39, 40]. First, a qualitative/narrative synthesis will be completed (e.g., synthesize primary studies to explore heterogeneity descriptively through structured narratives, summary tables, or figures to aid in data presentation where appropriate). Details of intervention paradigms (e.g., procedures, recipients, provider) may be summarized in a table per the Template of Intervention Description and Replication guidance [41].

Next, study data will be quantitatively synthesized, if there is an alignment in the outcome measures for the primary and secondary outcomes (e.g., self-reported or proxy reported ratings of sleep quality on the same scale).

If the data is appropriate for quantitative synthesis, studies will be pooled in statistical meta-analysis (e.g., random effects model) using JBI SUMARI. Effect sizes will be expressed as either odds ratios (for dichotomous data) and weighted (or standardized) final post-intervention mean differences (for continuous data) and their 95% confidence intervals will be calculated for analysis. Dependent on the data available, planned summary measures (e.g., risk ratios, standardized mean differences) will be calculated. Heterogeneity will be assessed statistically using the standard chi-squared and I-squared tests. Statistical analyses will be performed using random effects [42]. Additional proposed subgroup analysis based on the findings includes but is not limited to analyses by cancer type, age groups (e.g., child versus adolescents), and treatment paradigms.

### Meta-biases

Sensitivity analyses will be conducted to test decisions made regarding sample size, methodological quality, or variance. A funnel plot will be generated to assess publication bias if there are 10 or more studies included in a meta-analysis. Statistical tests for funnel plot asymmetry (e.g., Egger test [43], Begg test [44], Harbord-Egger test [45]) will be performed where appropriate.

Further, studies will be classified based on the level of risk across several bias categories, including low, some concern, and high. Bias categories include selection bias (e.g., randomization, allocation concealment), performance bias (e.g., participant blinding to intervention arm), detection bias (outcome assessment blinding), attrition bias (e.g., attrition rate, use of intent-to-treat principle), reporting bias, and other bias sources.

### Assessing certainty in the findings

The Grading of Recommendations, Assessment, Development and Evaluation (GRADE) approach [46] for grading the certainty of evidence will be followed and a Summary of Findings (SoF) will be created using GRADEPro (McMaster University, ON, Canada). The SoF will present the following information where appropriate: absolute risks for the treatment and control, estimates of relative risk, and a ranking of the quality of the evidence based on the risk of bias, directness, heterogeneity, precision, and risk of publication bias of the review results. The outcomes reported in the SoF will include sleep quality, sleep disturbance, sleep duration, and daytime sleepiness.

## Discussion

This systematic review will synthesize and consolidate evidence on existing non-pharmacological interventions to improve sleep in pediatric cancer patients and survivors. A primary goal is to identify candidate interventions for standard of care recommendations based on efficacy and quality of evidence. Even if no strong recommendations for non-pharmacological sleep intervention for pediatric cancer patients and survivors emerge from this review, findings can still help inform clinical care. Aggregating relevant empirical interventions, their efficacy, and quality of evidence can help inform clinical decision-making even as standard of care recommendations are further researched and developed. In addition, the review may reveal innovative and emerging approaches to sleep intervention in this population that may have promise and may prove good candidates for further clinical investigation. Results of the review may identify effective interventions that are specific to certain settings, age groups, or other clinically relevant categorical criteria.

Beyond informing clinical care, the review is intended to identify gaps and opportunities to improve the methodical quality of further non-pharmacological sleep intervention research in this population toward developing an eventual standard of care. This review can offer researchers a head start in identifying and testing existing and new intervention paradigms to improve sleep, and subsequently quality of life, in pediatric cancer patients and survivors.

Several potential limitations exist that may impact this review. Publication bias and restriction of this review to English language publications may limit generalizability and robustness of relative recommendations for specific paradigms to improve sleep in pediatric cancer patients and survivors. Further, we recognize that there may be significant heterogeneity of intervention paradigms and outcome measures which may preclude statistical pooling and meta-analysis of findings. On the one hand, this is a potential limitation, though on the other being broadly inclusive of intervention paradigms may be useful in identifying innovative approaches to non-pharmacological sleep intervention that warrant further investigation. If meta-analysis is not feasible, the review will include a narrative synthesis of intervention effects. We will attempt to mitigate the impact of limitations through rigorous adherence to decision criteria and review methodology while highlighting potential limitations and cautious interpretation of findings.

The findings from this review will be disseminated through publication in a peer-reviewed journal.

## Supplementary Information

**Additional file 1.** PRISMA-P 2015 Checklist.

**Additional file 2.** Search strategy.

## Data Availability

Not applicable

## Abbreviations

JBI: Joanna Briggs Institute
JBI SUMARI: Joanna Briggs Institute System for the Unified Management, Assessment and Review of Information
SDPCM: Sleep Disturbances in Pediatric Cancer Model
PRISMA: Preferred Reporting Items for Systematic Reviews and Meta-Analyses
PRISMA-P: Preferred Reporting Items for Systematic Review and Meta-Analysis Protocols
PROMIS: Patient Reported Outcomes Measurement Information System
GRADE: Grading of Recommendations, Assessment, Development and Evaluation
