# Supplementary material for: Non-pharmacological sleep interventions for pediatric cancer patients and survivors: a systematic review protocol
Source: Syst Rev. 2021 Jun 4;10:166. doi: 10.1186/s13643-021-01724-3 (PMC8176735; doi:10.1186/s13643-021-01724-3)
Supplement: Supplementary file 2 — Additional file 2. Search strategy. [file 13643_2021_1724_MOESM2_ESM.docx]

## **Additional File 1: Search strategy**

Ovid Medline Search Strategy (all other included databases will be queried using a translated version of the same strategy).

1 exp *Sleep/

2 exp Sleep Wake Disorders/ or Fatigue/

3 exp "Sleep Initiation and Maintenance Disorders"/

4 (sleep* or insomnia*).ab,ti,kf.

5 1 or 2 or 3 or 4 [sleep terms]

6 exp *complementary therapies/

7 exp acupuncture therapy/

8 exp holistic health/

9 exp homeopathy/

10 exp integrative oncology/

11 exp mind-body therapies/

12 exp musculoskeletal manipulations/

13 exp naturopathy/

14 exp reflexotherapy/

15 exp movement/ or exp Yoga/

16 exp Relaxation Therapy/

17 exp Mindfulness/

18 exp Meditation/

19 exp Cognitive Behavioral Therapy/

20 exp Massage/

21 exp Breathing Exercises/

22 exp Medicine, Chinese Traditional/

23 exp Nutrition Therapy/

24 exp Aromatherapy/

25 exp Music Therapy/

26 (("non-pharmacologic*" or alternative or complementary or homeopath* or holistic) adj3 (medicine* or therap* or treatment*)).ab,ti,kf.

27 acupuncture.ab,ti,kf.

28 (yoga or mindfulness or relax* or massag* or meditat* or breath* OR movement OR exercis* OR "physical activ*").ab,ti,kf.

29 ("cognitive behavioral therap*" or CBT).ab,ti,kf.

30 (nutrition* or diet*).ab,ti,kf.

31 (music or sound or noise or light* or smell* or scent* or aroma*).ab,ti,kf.

32 or/6-31 [Interventions]

33 5 and 32 [Sleep terms AND Intervention terms]

34 exp *adolescent/ or exp *child/ or exp *infant/

35 exp *Pediatrics/

36 (child* or pediatric* or paediatric*).ti,ab,kf.

37 (adolescen* or teen*).ti,ab,kf.

38 ("young adult*" or AYA).ti,ab,kf.

39 34 or 35 or 36 or 37 or 38 [Pediatrics terms]

40 exp *Neoplasms/

41 (cancer* or neoplas* or carcinoma* or malignan* or oncolog* or leukemi* or leukaemi* or lymphoma* or Hodgkin* or NHL or Wilm* or Ewing* or glioma* or sarcoma* or neuroblastoma* or retinoblastoma* or rhabdomyosarcoma* or craniopharyngioma* or medulloblastoma* or chemotherap* or "stem cell transplant*" or radiation* or radiotherap*).ti,ab,kf.

42 40 or 41 [Cancer terms]

43 33 and 39 and 42 [Sleep terms + Intervention terms + Pediatrics terms + Cancer terms]

44 limit 43 to english language
